# In vitro and in vivo investigation of the capacity of mulberroside A to inhibit senescence

**DOI:** 10.1038/s41538-025-00559-9

**Published:** 2025-09-30

**Authors:** Yang Liu, Mingfei Chen, Cong Chen, Gao Huang

**Affiliations:** 1 College of Traditional Chinese Medicine, Guizhou University of Traditional Chinese Medicine, Guiyang, Guizhou China; 2 Laboratory animal institute, Guizhou University of Traditional Chinese Medicine, Guiyang, Guizhou China

**Keywords:** Senescence, Pharmacodynamics

## Abstract

Mulberroside A, naturally existent from the mulberry plant, is known for its diverse biological activities. Our research indicates that mulberroside A may possess significant anti-aging effects. We assessed its anti-aging properties using naturally aging animal models and by inducing senescence in human-derived endothelial cells and mouse-derived endothelial cells through treatment with angiotensin II. We found that mulberroside A promotes the proliferation of HMEC-1 and bEnd.3 endothelial cells, while significantly reducing the levels of senescence biomarkers p16, p21, and Rb in both these endothelial cell types and in the hippocampus, kidney, spleen and liver of naturally aging animals. Additionally, mulberroside A was found to mitigate telomerase depletion in the blood of naturally aging animals, enhance the body’s resistance ability to oxidative stress, and inhibit the overexpression of inflammatory factors in vivo.

## Introduction

Senescence is a physiological response to various stressors that impede the proliferation of senescent cells. The process of ageing initially manifests through cellular senescence and cycle arrest, morphological alterations, and the appearance of a senescence-associated secretory phenotype. Aging plays a significant role in the development of age-related diseases. The accumulation of senescent cells may contribute to the deterioration of multiple organ functions^1^. Aging involves genomic mutations, insufficient telomere length, epigenetic alterations, protein degradation, immune system decline, and chronic inflammation. SASP triggers senescence in precancerous cells, yet these cells are subsequently eliminated by immune surveillance to prevent early carcinogenesis^2^. The secretion of SASP by senescent cells leads to the development of aseptic chronic inflammation, which exerts significant impact on the surrounding microenvironment^3^. In the process of aging and the development of age-related diseases, cells exhibiting markers of senescence accumulate in various tissues. Concurrently, the efficiency of the immune system in clearing these senescent cells diminishes with advancing age^4^. The extraction of natural products with broad nutritional effects from natural resources has practical significance. For instance, plants such as *Panax ginseng* C.A. Meyer, *Astragalus membranaceus* (Fisch.) Bge. var. *mongholicus* (Bge.) Hsiao and *Ganoderma lucidum* are rich in bioactive compounds, including polysaccharides, polyphenols, and vitamins. These bioactive compounds have been shown to effectively modulate a variety of physiological processes, such as antioxidation, anti-inflammation, telomere maintenance, and mitochondrial repair, consequently delaying the onset of age-related diseases^5,6^. These compounds possess dual functionalities in both food and medicine, demonstrating significant potential for maintaining the health of the elderly population and extending their healthy lifespan.

Mulberry, known as *Morus alba L*. in Latin, is an ancient tree species that has been cultivated for over a millennium and has been extensively utilized across East Asia. The fruit of the mulberry is consumed for its various health benefits. Meanwhile, the leaves can be ingested or used medicinally. Moreover, the processed bark from its roots has therapeutic properties. Mulberry contains bioactive compounds that have shown potential anti-inflammatory effects and regulation of obesity and diabetes. They also possess antibacterial properties and act as antioxidants while improving vision and managing hypertension by modulating cholesterol transport, among other beneficial effects. Despite extensive preliminary research on mulberry extracts, the specific natural compounds that confer their anti-aging properties have yet to be identified^7^. It is of great significance to further explore the biological activities of mulberroside A and reinforce fundamental research. Mulberroside A, a naturally occurring compound found in mulberry, has been shown to alleviate osteoarthritis and mitigate intervertebral disc degeneration^8^. The phenolic hydroxyl groups present in its derivatives enable it to exert skin-whitening effects by inhibiting tyrosinase activity^9,10^. Moreover, mulberroside A exhibits potential therapeutic effects on neurodegenerative disorders, such as Parkinson’s disease. However, whether mulberroside A possesses additional biological activities remains to be elucidated. Therefore, our study focuses on investigating and validating the anti-aging effects of mulberroside A.

## Results

### Analysis of mulberroside A effects on cell growth

**Fig. 1:**
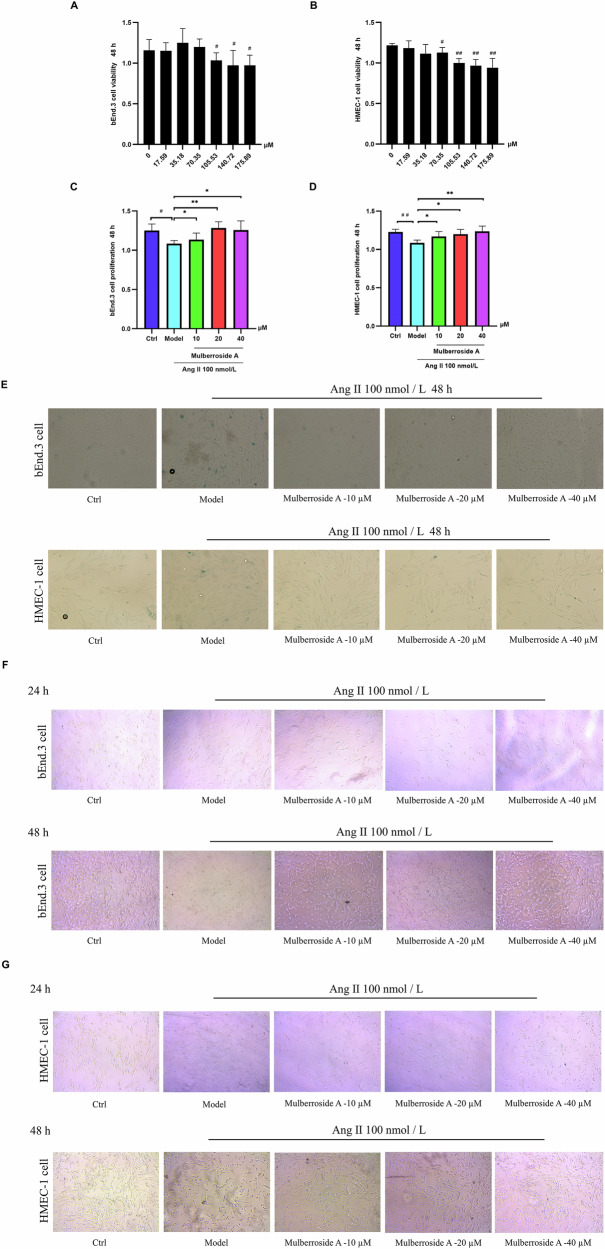
To evaluate the inhibitory and proliferative effects of mulberroside A on HMEC-1 and bEnd. 3 cells, we utilized the CCK-8 assay to measure cell proliferation activity. Cells were exposed to 100 nmol/L Ang II or different concentrations of mulberroside A for 48 h to evaluate their respective effects on cell proliferation and inhibition. Additionally, SA-β-Gal staining was employed to assess cellular senescence, revealing that mulberroside A effectively inhibited cell senescence. **A** Modulation of bEnd.3 cells activity by mulberroside A. **B** Investigation of the regulatory effects of mulberroside A on the activity of HMEC-1 cells. **C** The prominent proliferative effect of mulberroside A on bEnd. 3 cells. **D** The prominent proliferative effect of mulberroside A on HMEC-1 cells. **E** SA-β-Gal staining. The cells in a senescent state exhibit a blue staining reaction. **F**, **G** The number density of the two cell types as observed under a conventional optical microscope (^#^*P* < 0.05 vs. Ctrl group, ^# #^*P* < 0.01 vs. Ctrl group; ^*^P < 0.05 vs. Ang II group, ^* *^P < 0.01 vs. Ang II group).

The renin-angiotensin-aldosterone system (RAAS) significantly influences longevity regulation in diverse organisms. Its primary effector, angiotensin II, induces oxidative stress through reactive oxygen species production, hastening vascular aging processes. Age-related vascular dysfunction is closely associated with senescent endothelial cell accumulation, which serves as a pathogenic factor in age-associated vascular disorders^11,12^. Therefore, we chose both human and murine vascular endothelial cells as model systems to systematically investigate the anti-aging effects of mulberroside A. Following a 48-hour co-culture of mulberroside A with bEnd.3 and HMEC-1 cells, cytotoxic effects were observed at concentrations greater than 70.35 µM. Based on these findings, we selected concentrations of mulberroside A for further investigations, specifically 10 µM, 20 µM, and 40 µM, for both cell types. After co-cultivating mulberroside A with bEnd.3 and HMEC-1 cells for 48 h, we determined that the optimal concentrations of mulberroside A were 10 µM, 20 µM, and 40 µM for both cell types (Fig. 1A, B). According to the current data, cellular senescence was evident in cells treated with Ang II for 48 h^13^. When Ang II was co-incubated with the cells, it markedly suppressed the proliferative activity of both HMEC-1 and bEnd. 3 cells. However, as the concentration of mulberroside A increased, mulberroside A progressively mitigated the inhibitory effect of Ang II, leading to a dose-dependent recovery in the proliferative activity of both cell types (Fig. 1C, D). The presence of positive SA-β-gal staining serves as a hallmark indicative of cellular senescence^14^. We re-stained the two cell populations with SA-β-Gal staining to evaluate the effect of mulberroside A on their proliferation. In the 48 h experiment, the number of cells showing a blue reaction is significantly higher in the Ang II-exposed group compared to the control group that is not exposed to Ang II, suggesting an exacerbation of the aging phenomenon. Meanwhile, our findings indicate that the concentration of mulberroside A exhibits a negative correlation with the proportion of cells displaying a blue reaction. This suggests that as the concentration of mulberroside A increases, the number of cells exhibiting a blue reaction decreases, thereby implying that mulberroside A may possess the potential to reduce the population of senescent cells (Fig. 1E). In the 48 h experiment, visual inspection reveals that the number of bEnd. 3 and HMEC-1 cells exposed to Ang II for 48 h is significantly lower compared to the control group not exposed to Ang II, indicating inhibited growth activity. Additionally, as the concentration of mulberroside A gradually increased during the cell growth process, a significant increase in the number of bEnd. 3 and HMEC-1 cells is observed (Fig. 1F, G). In summary, the research findings suggest that mulberroside A may have a promoting effect on cell proliferation, and further observation and verification will be conducted.

### The EdU assesses the growth-promoting ability of mulberroside A on cells

**Fig. 2:**
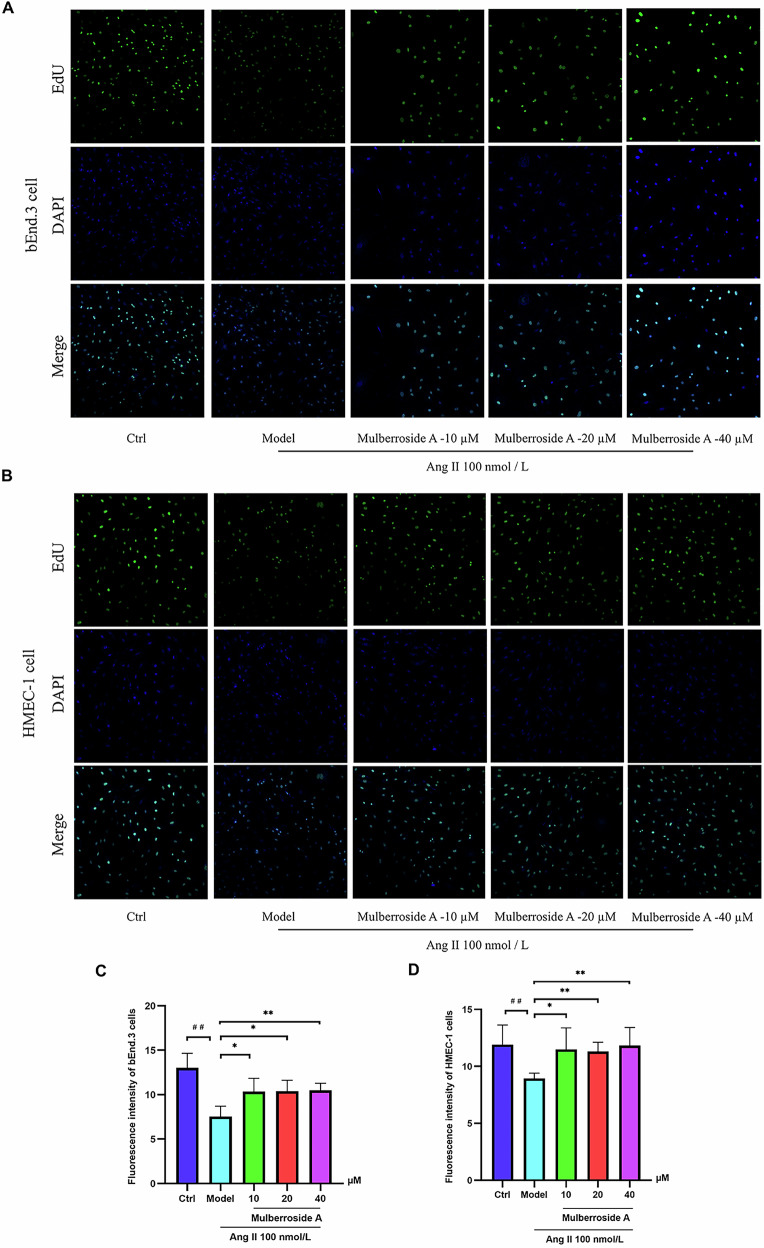
The proliferative effect of mulberroside A on bEnd.3 and HMEC-1 cells is evidenced by the presence of intense green fluorescence. **A**, **C** The effect of mulberroside A on the proliferation capacity and fluorescent activity of bEnd.3 cells at different concentrations. **B**, **D** The regulatory effect of mulberroside A treatment on the proliferation activity and fluorescence signaling in HMEC-1 cells (^#^*P* < 0.05 vs. Ctrl group; ^*^P < 0.05 vs. Ang II group, ^**^P < 0.01 vs. Ang II group).

In heterogeneous cell populations, individual proliferating cells can be precisely detected using 5-ethynyl-2’-deoxyuridine staining technology, as cells in an actively dividing state will exhibit distinct and unique fluorescence^15^. We utilized this method to assess the proliferative effects of mulberroside A on senescent HMEC-1 and bEnd.3 cells. Following the addition of Ang II to the culture medium for both cell types, active cells rapidly transitioned into a state of growth arrest, as evidenced by a significant reduction in the intensity of growth-associated fluorescence. However, when mulberroside A was co-administered with Ang II, the growth-arrested cells promptly transitioned into a proliferative state, demonstrating a substantial increase in the intensity of growth-associated fluorescence (Fig. 2).

### The impact of mulberroside A on aging-related biomarkers

**Fig. 3:**
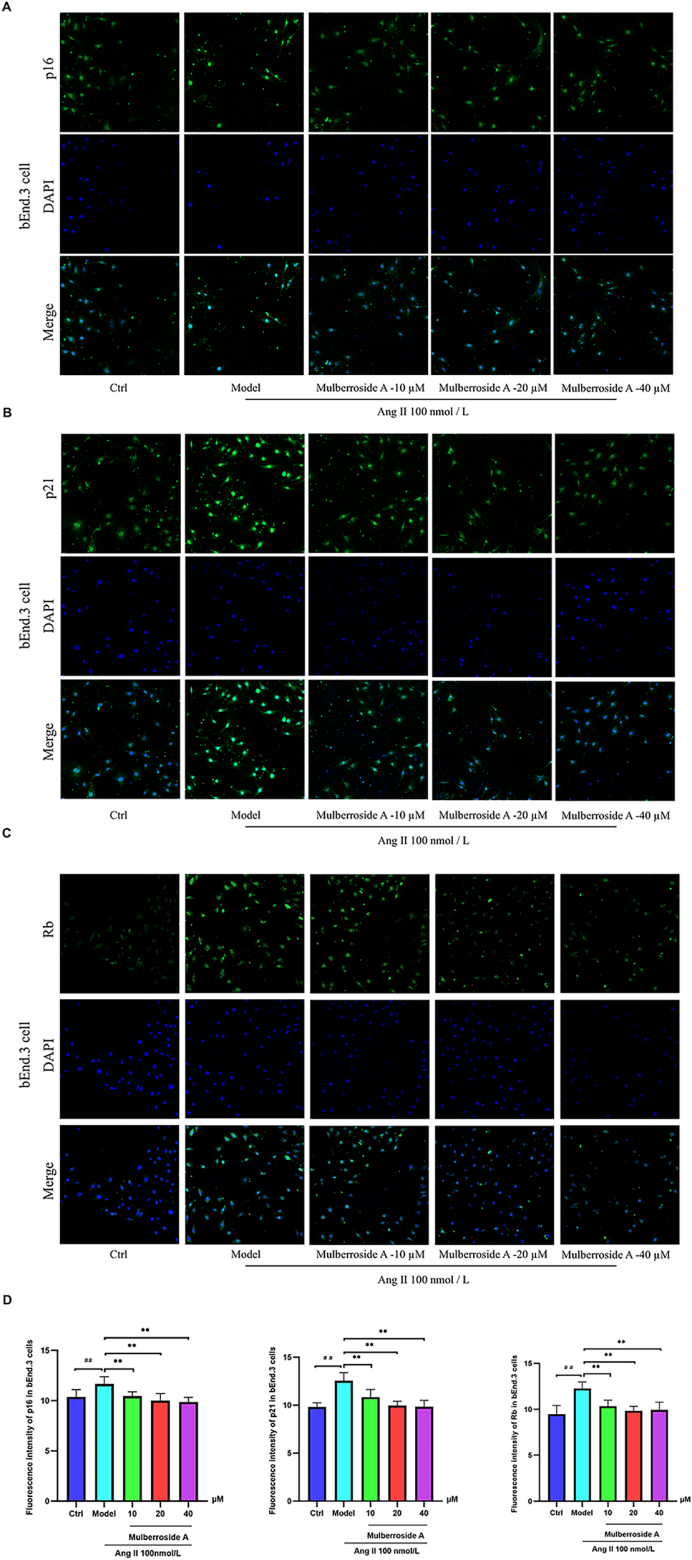
The fluorescence activity of the cells was analyzed. **A–D** Following treatment with different concentrations of mulberroside A, the expression levels of cell cycle regulatory proteins, including p21, p16, and Rb, were evaluated (^##^*P* < 0.01 vs. Ctrl group; ^**^P < 0.01 vs. Ang II group).

**Fig. 4:**
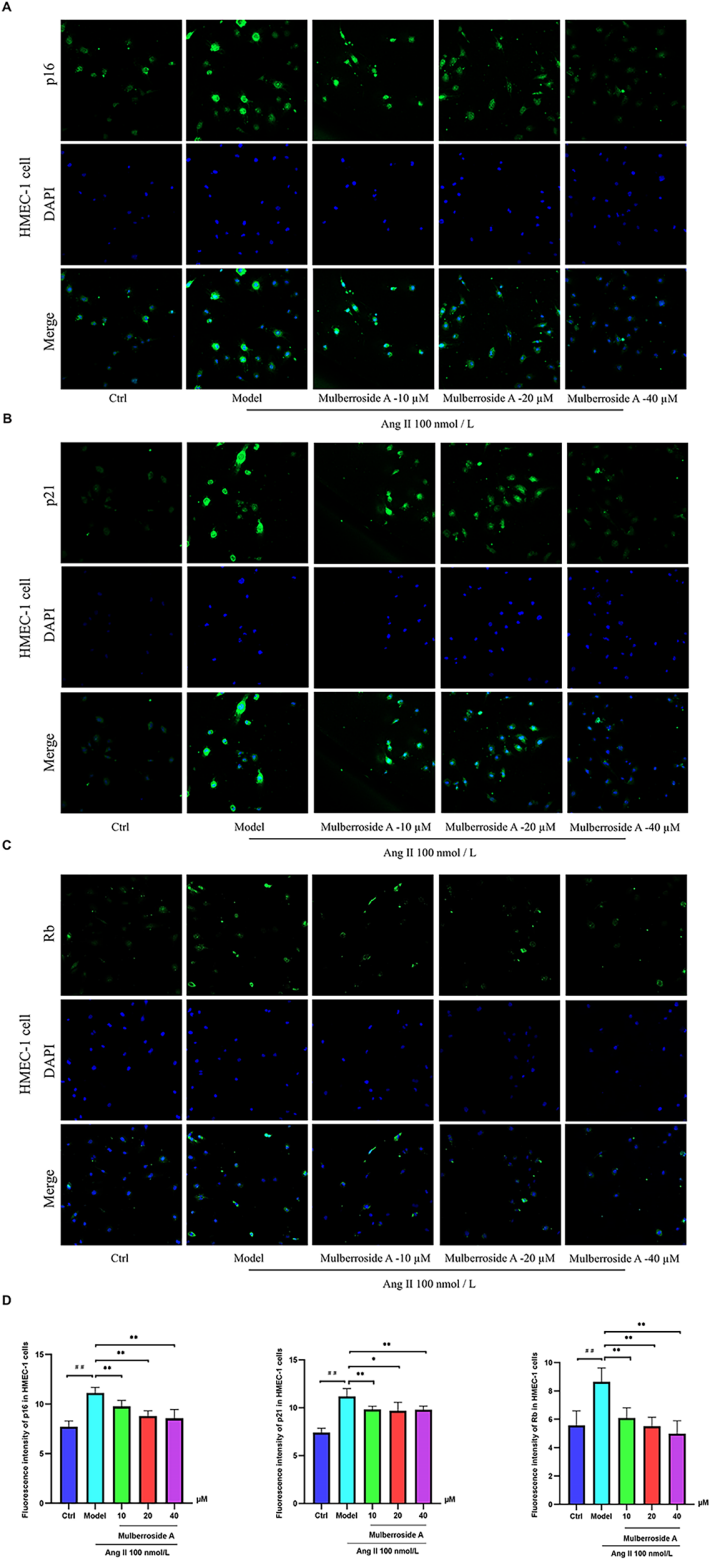
The fluorescence intensity of the cells was investigated. **A–D** The impact of mulberroside A on the expression levels of cellular senescence biomarkers p21, p16, and Rb in HMEC-1 cells (^##^*P* < 0.01 vs. Ctrl group; ^*^P < 0.05 vs. Ang II group, ^**^P < 0.01 vs. Ang II group).

Viral invasion or DNA damage leads to an increase in p21 levels, which promotes the formation of the Rb-E2F complex and subsequently suppresses the expression of cell cycle-related genes^16,17^. Removing high levels of p21 from cells can improve bodily function in the later stages of life and extend lifespan^18^. As a key molecular marker for identifying senescent cells, p16 maintains the non-phosphorylated state of Rb by specifically inhibiting the kinase activity of CDK 4 / 6, thereby blocking the critical regulatory point of G1 / S phase transition and ultimately triggering irreversible cell cycle arrest. This mechanism of action makes it one of the core indicators for assessing the aging process of tissues^19^. Treatment of bEnd.3 and HMEC-1 cells with Ang II resulted in cellular senescence, characterized by a significant elevation in the levels of senescence-associated biomarkers p21, p16, and Rb. The gradual introduction of mulberroside A effectively attenuated the expression of these biomarkers (Figs. 3, 4).

### The regulatory effects of morinidazole A on the ethology and morphology of naturally aging model

**Fig. 5:**
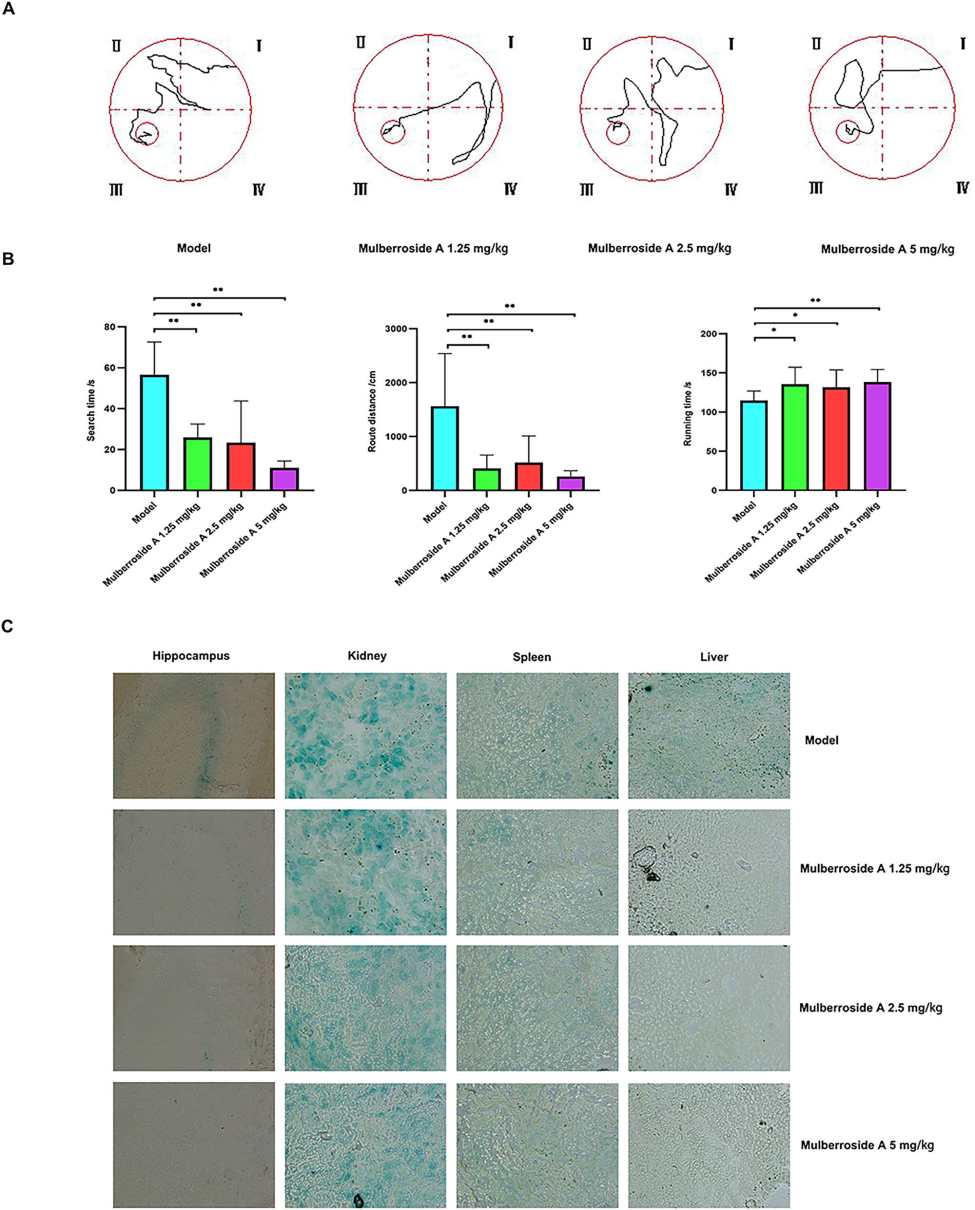
Effect of mulberroside A on ethology and morphology of naturally aging model. **A** The behavioral performance of animals in the Morris maze. **B** The ethology results, presented from left to right, encompass the search time, route distance, and running time. **C** The SA-β-Gal staining of frozen sections of hippocampus, kidney, spleen, and liver.

The time required for naturally aging mice to locate the platform and the distance traveled significantly increased, while their endurance during running was markedly reduced. Compared with the naturally aging group, mice administered varying doses of mulberroside A exhibited significantly shorter latency in locating the platform and reduced travel distances within the water maze, while demonstrating prolonged durations of movement (Fig. 5A, B). Figure 5C clearly shows that the hippocampus, kidney, spleen, and liver of naturally aging animals exhibit obvious blue reactions. When treated with different doses of mulberroside A, the blue reactions in these organs became significantly less pronounced.

### Mulberroside A reduces the expression of aging biomarkers in naturally aging animals

**Fig. 6:**
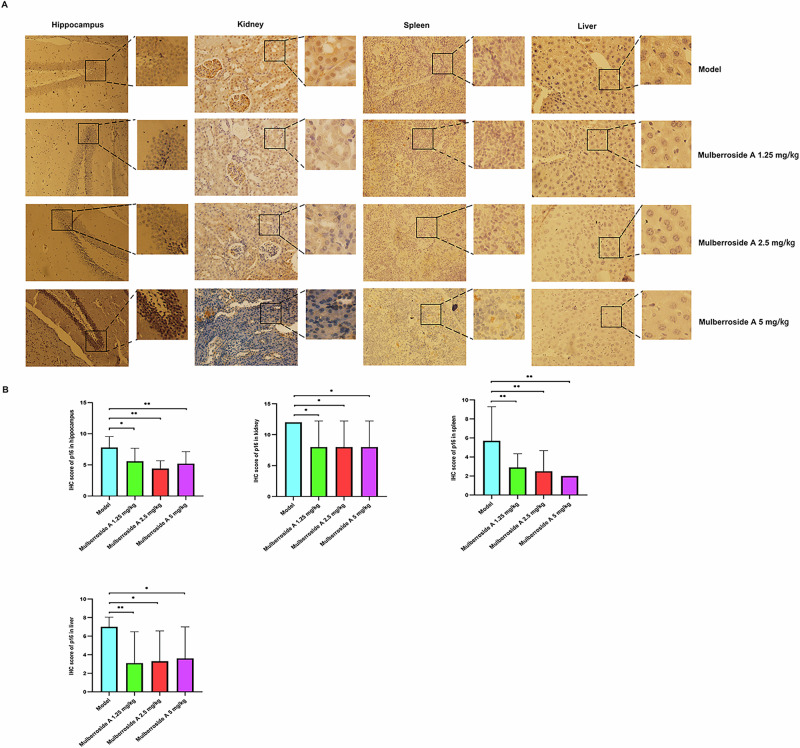
Effect of mulberroside A on the organ aging biomarker p16 in naturally aging animal model. **A** Immunohistochemical staining images. **B** IHC score.

**Fig. 7:**
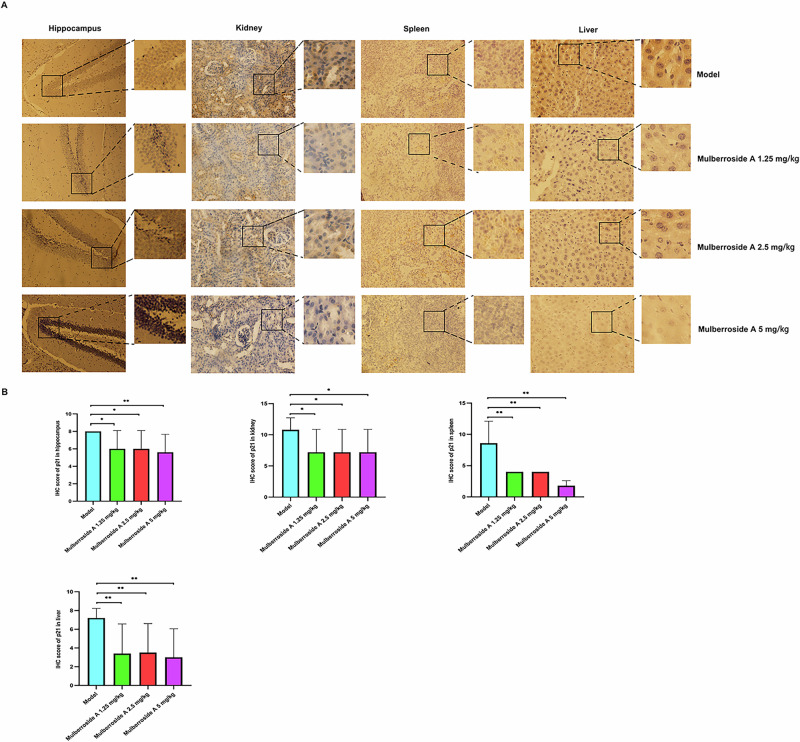
Effect of mulberroside A on the organ aging biomarker p21 in naturally aging animal model. **A** Immunohistochemical staining images. **B** IHC score.

**Fig. 8:**
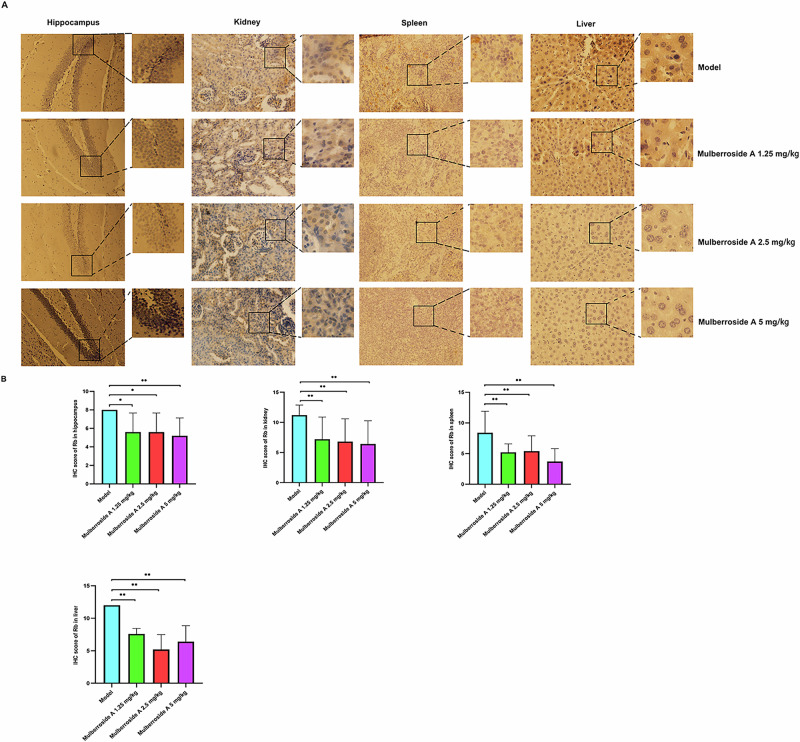
Effect of mulberroside A on the organ aging biomarker Rb in naturally aging animal model. **A** Immunohistochemical staining images. **B** IHC score.

During the physiological aging process, the protein expressions of p16, p21 and Rb in the hippocampus, kidney, spleen and liver tissues of animals show significant age-related upregulation. However, the following treatment with gradient concentrations of mulberroside A resulted in progressively decreasing levels of these biomarkers in the aforementioned organs. This suggests that mulberroside A is capable of effectively inhibiting the expression of aging-related biomarkers (Figs. 6–8).

### Mulberroside A modulates plasma biochemical parameters in naturally aging animals

**Fig. 9:**
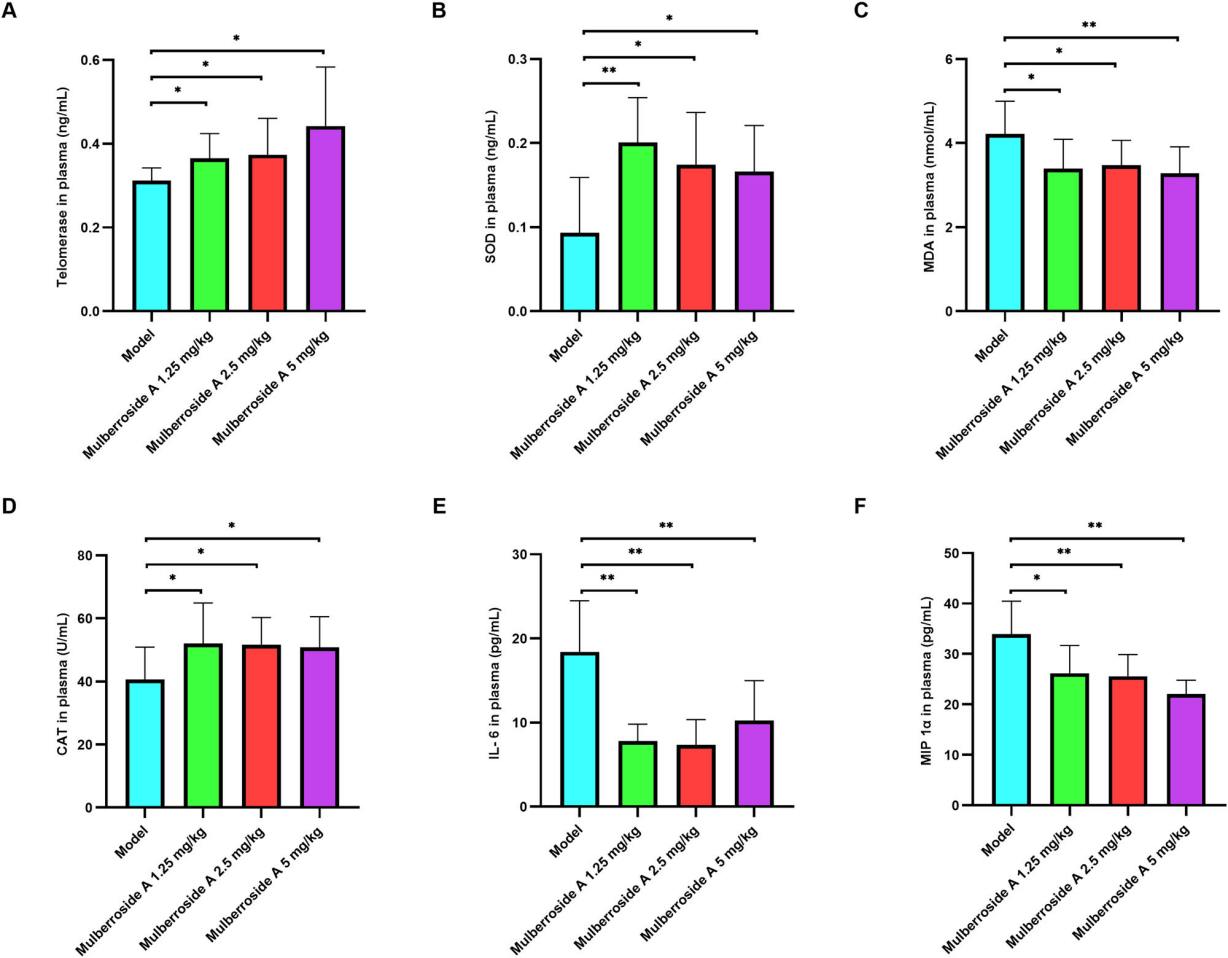
The effect of mulberroside A on SASP factor in plasma. Regulation of different concentrations of mulberroside A on telomerase, superoxide dismutase (SOD), catalase (CAT), malondialdehyde (MDA), interleukin 6 (IL-6), macrophage inflammatory protein 1α (MlP 1α) in plasma of naturally aging animals. **A** The telomerase in plasma. **B** The SOD in plasma. **C** The MDA in plasma. **D** The CAT in plasma. **E** The IL-6 in plasma. **F** The MIP 1α in plasma.

Figure 9 shows that the levels of telomerase, SOD, and CAT in the plasma of naturally aging animals were significantly lower than those in the intervention groups treated with gradient doses of mulberroside A. Meanwhile, the contents of MDA, IL-6, and MIP-1α in the plasma of naturally aging animals were significantly higher than those in the intervention groups treated with gradient doses of mulberroside A. These results suggest that mulberroside A can inhibit telomerase loss induced by aging, enhance the body’s resistance ability to oxidative stress, and suppress the overexpression of inflammatory factors.

## Discussion

As time progresses, the capacity of tissues to recover from stress gradually diminishes, leading to an elevated risk of various degenerative diseases. Aging, as a cellular fate process, is characterized by the loss of proliferative potential in normally replicating cells and the development of SASP, which includes pro-inflammatory cytokines and chemokines, as well as tissue-damaging proteases^20^. Aging biomarkers encompass elevated expression levels of cell cycle-dependent kinase inhibitors such as p16 and p21, increase production of SASP factors including IL-6, MIP and TNFα, and enhance activity of SA-β-gal^21^. The methods for extending healthy lifespan include adopting a rational lifestyle and implementing appropriate dietary interventions. Plant metabolites with nutritional functions are increasingly recognized for their potential to prevent aging and promote healthy longevity^22^. Mulberroside A is a bioactive compound found in the dietary supplement mulberry and has been shown to possess anti-inflammatory, antitussive, and diuretic properties. However, the current body of evidence is inadequate to conclusively validate its anti-aging effects. Therefore, we have developed both cellular and animal models of aging to investigate the potential anti-aging efficacy of mulberroside A. We induced senescence in human and murine endothelial cells using Ang II. Senescent cell was quantified via SA-β-Gal staining, cellular proliferation was assessed by EdU incorporation, and the expression levels of senescence-associated biomarkers were evaluated through immunofluorescence staining. These experiments reveal that mulberroside A significantly ameliorates cellular senescence and enhances proliferative capacity. We know vascular endothelial cell senescence acts as both an early indicator and a pivotal mechanism underlying vascular aging in animals. Senescent endothelial cells display reduced regenerative potential, increased secretion of inflammatory mediators, mitochondrial dysfunction, and impaired oxidative stress responses^23,24^. These changes directly contribute to vascular dysfunction and expedite systemic aging processes. The vascular system transports oxygen-rich and nutrient-rich blood to various organs via arterial branches, while capillary networks facilitate substance exchange^25,26^. Cerebral tissue exhibits an extraordinary dependence on a continuous blood supply, as cerebrovascular dysfunction can directly result in neuronal injury and cognitive impairment. The blood–brain barrier endothelial cells maintain low permeability through tight junctions, while liver sinusoidal endothelial cells possess fenestrated structures to enable efficient macromolecular exchange^27,28^. We investigated the effects of mulberroside A on senescence biomarkers and the SASP in naturally aging animal model. Our findings confirm its potent inhibitory effect on senescence biomarkers, including p16, p21, and Rb, across multiple organs such as the hippocampus, kidney, spleen, and liver. Further analysis of blood biomarkers demonstrates that mulberroside A effectively mitigates telomerase depletion, enhances the activities of antioxidant enzymes such as SOD and CAT, reduces MDA levels, and suppresses the elevation of inflammatory factors, including IL-6 and macrophage inflammatory protein. Collectively, both in vitro and in vivo data demonstrate that mulberroside A effectively modulates the overexpression of senescence biomarkers. However, this study also has certain limitations and areas for further exploration. During the aging experiment, there was a lack of in-depth analysis and validation regarding the anti-aging targets of mulberroside A. In this study, to minimize potential confounding factors related to secondary pathologies associated with advanced age, only naturally aged animals that have not yet reached the advanced stage of senescence were selected. Although we have demonstrated the anti-aging effect of mulberroside A through animal or cell experiments, it is important to note that experimental conditions involving animals and cells are generally more controllable than those involving humans. The aging process in humans, along with the mechanisms that regulate lifespan, is highly complex. Various biomarkers of aging are currently being investigated in therapeutic strategies focused on preserving health and promoting longevity. Investigating the impact of these interventions on the multimodal interactions among aging mechanisms will be crucial^29^. At present, it is impossible to completely reverse the aging process. What can be achieved is merely a delaying effect. The research on it is a long-term and complex task. Further extensive exploration and study are of vital importance for advancing the understanding of aging. In sum-up, mulberroside A significantly inhibits Ang II-induced cellular senescence, promotes the proliferation of both human and murine endothelial cells, and mitigates alterations in aging biomarkers within organs of naturally aging animals. By modulating the p16/p21/Rb signaling in naturally aging model organisms and senescent cells, these findings offer valuable insights into the anti-aging mechanisms of mulberroside A.

## Methods

### Reagents

Mulberroside A, >98% purity, bought from DESITE Biotechnology Co., Ltd (Chengdu, China). Vascular endothelial cells derived from mouse (bEnd.3) and human (HMEC-1) were received from National Collection of Authenticated Cell Cultures (Shanghai, China). Mouse IL-6 ELISA (SEKM-0007), catalase activity (BC0205), malondialdehyde content (BC0025), and senescence-associated β-galactosidase staining (G1580) kits were obtained from Solarbio (Beijing, China). MCDB 131 medium (PM 151210) and DMEM medium (PM 150210B) were accepted from Pricella (Wuhan, China). Angiotensin Ⅱ (GP 10023) was acquired from GLPBIO (Shanghai, China). Additional ELISA kits included mouse telomerase and macrophage inflammatory protein 1α from CUSABIO (Wuhan, China), and superoxide dismutase (MES134Mu) from Cloud-Clone Corp (Wuhan, China). Primary antibodies targeting CDKN2A/p16-INK4a (bs-23797R), CDKN1A/p21 (bs-0741R), and Rb (bs-2777R) were acquired from Bioss (Beijing, China).

### Cells culture

Vascular endothelial cells, specifically the murine bEnd.3 and human HMEC-1 lines were cultured in DMEM or MCDB 131 medium supplemented with 10% fetal bovine serum. The culture medium was refreshed immediately upon the cells reaching 80% confluence. Following this, the cells were detached using trypsin and subsequently incubated at 37 °C in a 5% CO_2_ incubator for further cultivation.

### Cells viability assay

The bEnd.3 or HMEC-1 cells were seeded in 96-well plates at 5 × 10^3^ cells per well. After 24 h, mulberroside A was added at concentrations of 0, 17.59, 35.18, 70.35, 105.53, 140.72, and 175.89 µM. Cells were initially cultured under standard conditions (37 °C, 5% CO₂) for 48 h, followed by the addition of 10 µL of CCK-8 reagent per well and a subsequent 4-hour incubation. The absorbance at 450 nm was measured to determine the optimal drug concentration. For subsequent experiments, cells were replated at 5 × 10³ cells/well and co-exposed to mulberroside A and 100 nmol / L angiotensin II for an additional 48 h under identical culture conditions. After this period, 10 µL of CCK-8 solution was added, followed by a 4 h incubation in the dark. Absorbance at 450 nm was measured using Thermo FC K 3 microplate reader (Shanghai, China).

### Senescence-associated β-galactosidase (SA-β-Gal) staining assay

Cells were seeded at 1 × 10⁵ cells / well in 6-well plates and cultured to confluence. Following medium removal, plates were washed once with phosphate-buffered saline (PBS), then treated with 1 mL β-galactosidase fixing solution per well for 15 minutes at room temperature. After fixation, wells were supplemented with staining solution (100 mM sodium phosphate, 2 mM MgCl₂, 150 mM NaCl, 0.01 % sodium deoxycholate, 0.02 % NP-40, 5 mM each potassium ferricyanide / ferrocyanide, and 1 mg / mL X-gal) and incubated overnight at 37 °C. Stained cells were visualized using an OLYMPUS CX33 microscope with random field selection (Shenzhen, China).

### EdU staining assay

The 5 × 10^3^ cells were cultured on round glass sheet. Solution of 10 μM 5- ethynyl-2’-deoxyuridine was added into the wells and incubated for 2 h. The cells were fixed using 200 μL of a 4% paraformaldehyde solution for 15 min. Subsequently, the glass sheet underwent washing with an appropriate rinsing liquid. The cell nuclear were stained with Hoechst 33342 for up to 10 min. The cellular imagery was meticulously acquired by means of fluorescence microscopy. Data are automatically acquired after capturing five random sample images using the ZEISS Vision Care fluorescence microscope (Shanghai, China).

### Immunofluorescence assay

Cells were fixed with 4% paraformaldehyde for 30 min, permeabilized with 1% Triton X-100 for 10 min, and blocked with goat serum. They were then incubated overnight at 4° C with p16, p21, and Rb antibodies (1:100). Subsequently, the cells were incubated with goat anti-rabbit IgG (1:1000) for 1 h under dark conditions at room temperature. Data are automatically acquired after capturing five random sample images using the ZEISS Vision Care fluorescence microscope (Shanghai, China).

### Naturally aging model

The SPF male C57BL/6J mice, purchased from Tengxin Biotechnology Co., Ltd (Chongqing, China), bred by the Animal Laboratory Center of Guizhou University of Traditional Chinese Medicine. Forty 13-month-old C57BL/6J mice were divided into four groups: a naturally aging control group with 10 subjects, and a mulberroside A gradient dose orally group with 30 subjects (1.25, 2.5, and 5 mg/kg). After continuous administration for 12 weeks, animal behavior was monitored, and samples of plasma, spleen, liver, kidney, and hippocampus were collected. Throughout the research period, the animals were housed under standard laboratory conditions with unrestricted access to food and water as well as freedom of movement. All experimental procedures were carried out in accordance with institutional guidelines and relevant regulatory standards. Upon completion of the experiment, the animals were deeply anesthetized with nebulized isoflurane gas for euthanasia. The research protocol was formally approved by the Animal Ethics Committee of Guizhou University of Traditional Chinese Medicine (No. 2024116).

### Morris maze navigation experiment

Mice were introduced into the pool at the starting point, and the latency to locate the platform was measured. If mice failed to find the platform within 90 s, the escape latency was recorded as 90 s. The training was conducted over a continuous 6-day period. On the 7th day, the mice were placed in the pool at the designated starting point. The following metrics were recorded: the time taken for the mice to reach the platform for the first time within 90 s, and the total distance traveled along their trajectories.

### Running experiment

Using an animal treadmill, the running duration was adjusted to 30 min, the electrical stimulation intensity was set to 0.8 mA, and data recording was terminated when the mouse ceased running following three consecutive episodes of electrical stimulation.

### Frozen section

Freshly harvested tissues undergo rapid snap-freezing in liquid nitrogen prior to archival storage at -80 °C. For sectioning, the cryo-cut microtome chamber temperature is equilibrated to -20 °C. Tissues are subsequently embedded in OCT cryoprotectant (a polyvinyl alcohol / polyethylene glycol compound), then mounted onto the cryo-cut microtome stage using a specimen disc for stabilization. Tissue sections with a thickness of 10–15 μm were prepared using cryo-cut microtome and stained with SA-β-Gal (refer to the previous content for the specific method). The activity of senescence-associated β-galactosidase was analyzed using an OLYMPUS CX33 optical microscope (Shenzhen, China).

### Immunohistochemical method

The samples were dewaxed in xylene for 20 min, dehydrated in anhydrous ethanol for 5 min, and subsequently hydrated using a graded ethanol series, with each step lasting 2 min. After antigen retrieval in sodium citrate buffer for 10 min, the samples were incubated with blocking solution at room temperature for 60 min. Then, the samples were co-incubated with p16 antibody (1:100), p21 antibody (1:100), and Rb antibody (1:100) for 24 h. The samples were rinsed three times with PBS. HRP-conjugated secondary antibody (1:200) was added, and the mixture was incubated at room temperature for 1 h. The samples were stained using DAB chromogenic solution. Subsequently, they were counterstained with hematoxylin for 5 min, rinsed with running water for 15 min to allow for differentiation and color development, and finally dehydrated and cleared through a graded ethanol series of 70%, 80%, 90%, and 100% concentrations, with each step lasting 10 s. Finally, xylene was used to permeate the slices over 5 min prior to microscope observation after they were sealed with neutral resin. The staining intensity of the cells in the image and the proportion of positive cells were assessed and scored. These two scores were then multiplied to calculate the final result.

### Enzyme-linked immunosorbent assay

Follow the optimized operational guidelines provided with the kit, and use the key reagents that are either pre-configured or accurately labeled with their respective concentrations, including the standard solution, enzyme conjugate, substrate solution, and washing buffer. Standard wells are established to construct the standard curve, sample wells are designated for measuring the target analyte in plasma samples, and control wells are included to monitor the performance of the experimental system on the microplate. Standard apertures were sequentially added with 100 μL standard products of different concentrations. The blank well was added with 100 μL standard diluent. The remaining apertures were filled with the samples. The 96-well plate was incubated at 37° C for 1 h. Then, the test solution was mixed into the plate. After discarding the liquid in each well, all wells were washed three times with 350 μL scrubbing solution and tapped on absorbent paper to remove any remaining liquid. Finally, the OD values of telomerase, superoxide dismutase (SOD), catalase, malondialdehyde (MDA), interleukin 6 (IL-6), macrophage inflammatory protein 1α (MIP 1α) in each sample were immediately measured by microplate reader at a wavelength of 450 nm.

### Statistical analysis

The data analysis was conducted using SPSS 23.0 software, and the generation of visual outputs was achieved through GraphPad Prism 9.0. Non-parametric analysis was applied to trial data, with statistical significance set at *P* < 0.05.

## Data Availability

Data supporting the content of the study are included in the manuscript.
